# Unveiling the Gut Microbiota and Resistome of Wild Cotton Mice, *Peromyscus gossypinus*, from Heavy Metal- and Radionuclide-Contaminated Sites in the Southeastern United States

**DOI:** 10.1128/spectrum.00097-21

**Published:** 2021-08-25

**Authors:** Jesse C. Thomas, Troy J. Kieran, John W. Finger, Natalia J. Bayona-Vásquez, Adelumola Oladeinde, James C. Beasley, John C. Seaman, J. Vaun McArthur, Olin E. Rhodes, Travis C. Glenn

**Affiliations:** a Department of Environmental Health Science, University of Georgia, Athens, Georgia, USA; b Institute of Bioinformatics, University of Georgia, Athens, Georgia, USA; c Bacterial Epidemiology and Antimicrobial Resistance Research Unit, United States Department of Agriculture, Athens, Georgia, USA; d Savannah River Ecology Laboratory, University of Georgia, Aiken, South Carolina, USA; e Warnell School of Forestry and Natural Resources, University of Georgia, Athens, Georgia, USA; f Odum School of Ecology, University of Georgia, Athens, Georgia, USA; University of Auckland

**Keywords:** community structure, gut microbiota, heavy metals, metal resistance, antibiotic resistance, coselection, resistome

## Abstract

The prevalence of antibiotic resistance genes (ARGs) can be driven by direct selection from antibiotic use and indirect selection from substances such as heavy metals (HMs). While significant progress has been made to characterize the influence of HMs on the enrichment and dissemination of ARGs in the environment, there is still much we do not know. To fill this knowledge gap, we present a comprehensive analysis of gut bacteria associated with wild cotton mice (Peromyscus gossypinus) trapped from several areas affected by legacies of HM and radionuclide contamination. We explore how these contaminants affect gut microbial community (GMC) composition and diversity and the enrichment of antibiotic, biocide, and metal resistance genes. Although we were able to identify that a myriad of co-occurring antimicrobial and HM resistance genes appear in mice from all areas, including those without a history of contamination, the proportions of co-occurring ARGs and metal resistance genes (MRGs) are higher in sites with radionuclide contamination. These results support those from several previous studies and enhance our understanding of the coselection process, while providing new insights into the ubiquity of antimicrobial resistance in the resistome of wild animals.

**IMPORTANCE** Antimicrobial resistance is a serious global public health concern because of its prevalence and ubiquitous distribution. The rapid dissemination of antibiotic resistance genes is thought to be the result of the massive overuse of antibiotics in agriculture and therapeutics. However, previous studies have demonstrated that the spread of antibiotic resistance genes can also be influenced by heavy metal contamination. This coselection phenomenon, whereby different resistance determinants are genetically linked on the same genetic element (coresistance) or a single genetic element provides resistance to multiple antimicrobial agents (cross-resistance), has profound clinical and environmental implications. In contrast to antibiotics, heavy metals can persist in the environment as a selection pressure for long periods of time. Thus, it is important to understand how antibiotic resistance genes are distributed in the environment and to what extent heavy metal contaminants may be driving their selection, which we have done in one environmental setting.

## INTRODUCTION

Wildlife can act as ecological reservoirs for a variety of emerging and reemerging infectious diseases, many of which are zoonoses, a collection of diseases and infections naturally transmitted from vertebrate animals to humans (1). More than 1,500 pathogens, viral and bacterial, are known to infect humans and cause disease (2). According to the Centers for Disease Control and Prevention, more than 60% of these infectious diseases can be spread from animals to humans, and 75% of new or emerging infectious diseases in humans originate from animals (2, 3). Zoonotic bacterial diseases, in particular, can be transmitted from animals to humans through various modes of transmission: animal bites and scratches, direct fecal-oral route, and improper food handling, in addition to contaminated animal food products, soil, and water (2). Enteropathogenic bacteria such as Brucella, Campylobacter, *Clostridium*, Escherichia, *Listeria*, Salmonella, and *Shigella* species are among the most commonly reported bacterial pathogens transmitted by wild animals to humans, domestic stock, and pets (3, 4). Previous studies have identified the presence of these organisms in the gastrointestinal tracts and feces of a variety of wild animals (4–8). In addition, wild animals, including those that appear otherwise healthy, are frequently infested by a variety of blood-feeding arthropods (e.g., ticks, mites, etc.) that can transmit pathogenic or opportunistic species in the genera *Borrelia*, *Francisella*, *Rickettsia*, Treponema, and *Yersinia* (9).

Increasingly, considerable concern exists that wildlife can also act as reservoirs for antimicrobial-resistant (AMR) bacterial strains. Several reports describe clinically relevant bacterial strains detected in animal hosts (e.g., cattle, swine, rodents, bats, birds, etc.) harboring AMR genes associated with resistance to drugs commonly used for therapeutic purposes, such as β-lactams, aminoglycosides, quinolones, macrolides-lincosamides-streptogramin B (MLS), tetracyclines, and even last resort broad-spectrum antibiotics, such as carbapenems and vancomycin (4–8, 10–12). While antimicrobial resistance is a naturally occurring phenomenon in environments containing antibiotic-producing microorganisms (e.g., bacteria and fungi), current evidence suggests human activities have accelerated this process (13). For example, antimicrobial drugs originating from hospital and agricultural settings can contaminate terrestrial and aquatic habitats, where they can persist and select resistance in both host-associated and environmental bacterial strains (14, 15). This selective pressure can enrich antimicrobial resistance genes (ARGs), which can be rapidly disseminated and exchanged between bacteria via horizontal gene transfer of mobile genetic elements (MGEs) or through *de novo* mutations that are positively selected (16–18). In the intestinal tract of humans and animals, there is evidence that antibiotic use can enrich multiple ARGs in not only gut pathogens, but also commensals (4, 19–22).

In addition to antibiotics, there is evidence that the presence of heavy metals (HMs) in the environment can facilitate antibiotic resistance via coselection of ARGs and metal resistance genes (MRGs) (23–31). More recently, studies have begun to unravel the factors that influence the co-occurrence of ARGs and MRGs in complex microbial communities (32–34). While much of the existing literature describes the proliferation of antimicrobial resistance in natural and contaminated ecosystems, there are still large gaps in our understanding of the factors that influence the emergence, maintenance, and dissemination of ARGs and MRGs in wild animals. In this context, antimicrobial resistance is a global health problem, inasmuch as it is an ecological one. Furthermore, given the numerous interactions between humans and the environment, wildlife represent an important but often overlooked reservoir of multidrug-resistant bacteria (4, 8, 11, 35).

Previously, we used 16S rRNA gene amplicon sequencing and a shotgun metagenomic approach to investigate the soil microbiome in several areas at the Department of Energy’s Savannah River Site (SRS) affected by legacies of heavy metal (e.g., As, Cd, Cs, Co, Cr, Cu, Fe, Hg, Ni, Zn, U, etc.) and radionuclide (^137^Cs, ^60^Co, ^3^H, ^129,131^I, ^32^P, ^238,239,240^Pu, ^89,90^Sr, etc.) contamination (34, 36–38). We explored how these contaminants affect bacterial community composition and diversity and the enrichment of antibiotic, biocide, and metal resistance genes. Our investigations revealed these contaminants not only can have a profound impact on soil microbiome composition but also can affect how it functions. Here, we extend our analyses to include gut samples from wild Peromyscus gossypinus, the cotton mouse, which has an extensive range in the southeastern United States (39). These mice were trapped near the same GPS coordinates and time period as described in the aforementioned study (34, 40). We hypothesized that the gut microbial communities (GMCs) and associated antimicrobial and metal resistance determinants (i.e., the “resistome”) would differ in *P. gossypinus* mice from areas with different contaminant (i.e., metal and radionuclide) histories. Understanding the role of environmental contaminants in the coselection of ARG and MRGs in wild animal GMCs will aid in illuminating potential transmission of AMR from animals to humans.

## RESULTS

### Mouse trapping and sample collection.

**FIG 1:**
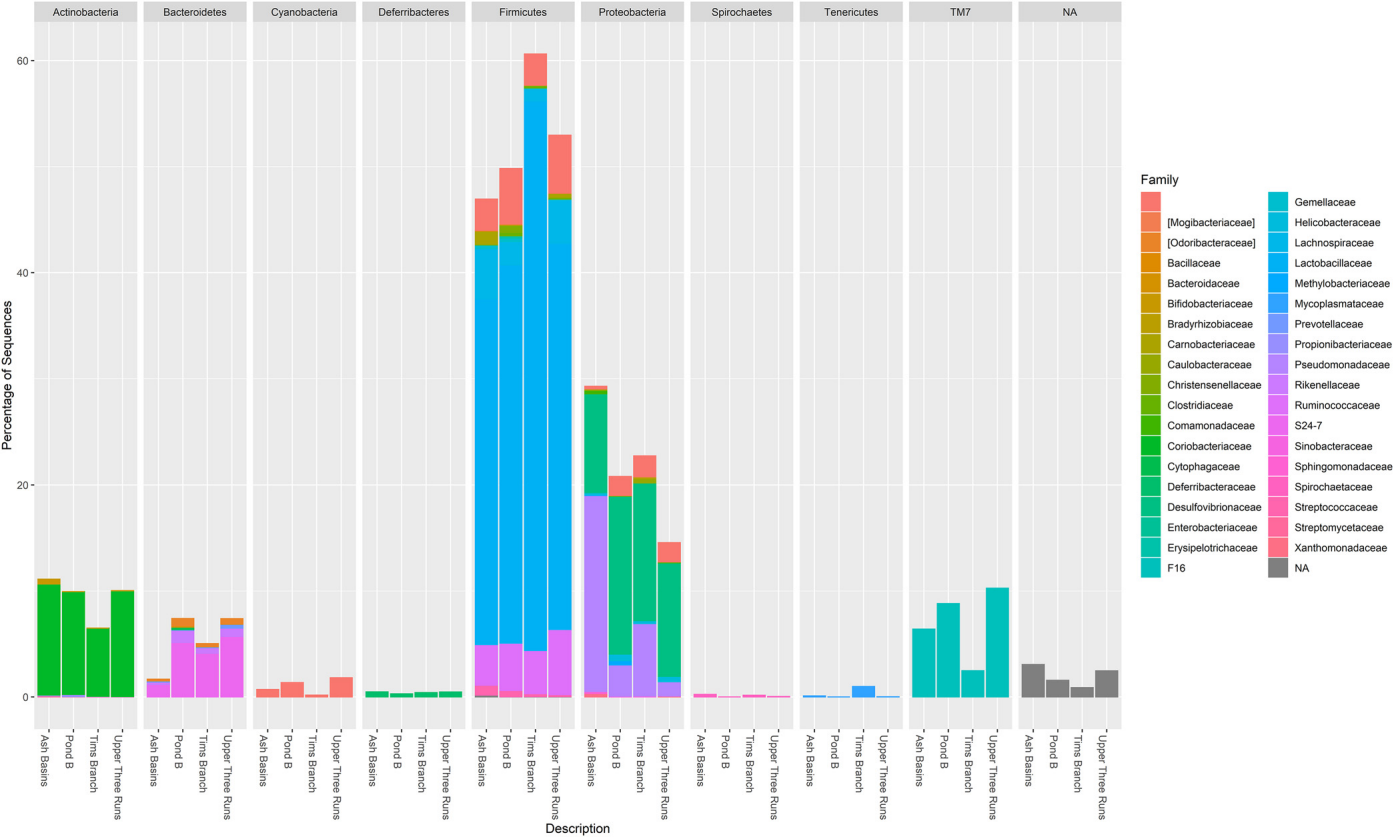
Relative abundance at the level of phylum and corresponding families representing the nine most abundant bacterial/archaeal OTUs (clustered at 97% similarity) in all *P. gossypinus* intestinal samples (small and large) from the four sampling sites. Bacterial phyla are further expanded into respective families.

The population of wild *P. gossypinus* mice examined in this study was previously collected by Tannenbaum and Beasley (40). Briefly, we used 86 wild mice (34 females, 52 males) captured from March to mid-May 2014 from four sampling sites at the SRS, SC, USA, characterized by differences in their contaminant profiles: uncontaminated (Upper Three Runs [UR]; *n *= 22), heavy metals (Ash Basins [AB]; *n *= 22), radionuclides (Pond B [PB]; *n *= 18), and both heavy metals and radionuclides (Tim’s Branch [TB]; *n *= 24) (Fig. 1; see Table S1a and b in the supplemental material) (40). Following dissection, intestinal samples (*n* = 171: small intestine, *n* = 86; large intestine, *n* = 85) were used for DNA extraction and 16S rRNA gene amplicon and shotgun metagenomic sequencing.

### Gut microbial composition and diversity based on 16S rRNA gene amplicon sequencing.

A data set of 7,712,212 high-quality 16S rRNA gene paired-end sequences from the 86 mice were generated with an average read length (± standard deviation [SD]) of 412.7 ± 29.2 bp. All subsequent analyses were performed on rarefied data using an even sampling depth of 15,000 reads per intestinal sample (*n *= 171) (Table S1a). A total of 90,862 operational taxonomic units (OTUs) (3,827 ± 2,035 OTUs/sample) were observed, spanning several bacterial phyla. Analysis of the core microbiome indicated there were 5 bacterial phyla (i.e., *Firmicutes*, *Proteobacteria*, *Actinobacteria*, *Bacteroidetes*, and TM7) shared between all the samples analyzed (Fig. 1).

Comparisons between the four sampling locations indicated significant differences (*P* < 0.05 is the significance threshold for all following tests) in *Bacteroidetes* (*P* = 5.81e−9), *Proteobacteria* (*P* = 2.80e−4), TM7 (*P* = 1.20e−5), *Verrucomicrobia* (*P* = 1.19e−4), and several others (see Table S2 in the supplemental material). While samples from AB had the highest mean (± SD) relative abundances of *Firmicutes* (46.00% ± 0.50%), *Proteobacteria* (26.85% ± 0.30%), and *Actinobacteria* (10.60% ± 0.20%) overall, pairwise group comparisons revealed AB samples had significantly lower abundance of *Bacteroidetes* compared to mice collected from PB and UR. Mice from TB had a significantly lower abundance of TM7 (*Saccharibacteria*), *Chlamydiae*, and *Cyanobacteria* than those from PB and UR (see Table S3 in the supplemental material). Relative abundances at the family level indicated there were several dominant bacterial families, including *Lactobacillaceae* (*Firmicutes*) (32.00% ± 8.49%), *Desulfovibrionaceae* (Deltaproteobacteria) (10.57% ± 1.95%), *Coriobacteriaceae* (*Actinobacteria*) (8.34% ± 1.51%), *Pseudomonadaceae* (*Gammaproteobacteria*) (6.25% ± 6.80%), *Ruminococaceae* (*Firmicutes*) (5.57% ± 1.17%), F16 (TM7) (5.45% ± 2.36%), S24-7 (*Bacteroidetes*) (4.90% ± 2.24%), and *Lachnospiraceae* (*Firmicutes*) (3.77% ± 1.30%). At the genus level, *Lactobacillus* and *Desulfovibrio* were the predominant genera across all intestinal samples. However, we observed that samples from AB and TB had significantly higher relative abundances of Pseudomonas and lower abundances of genera in the S24-7 family (*Bacteroidetes*), compared to either UR or PB (see Table S4 in the supplemental material). Regardless of sampling location, we also detected several potentially disease-causing infectious agents in addition to Pseudomonas in the gut tissues of these mice, including Acinetobacter, *Bartonella*, *Flexispira*, *Helicobacter*, Klebsiella, and *Yersinia* (Table S4).

**FIG 2:**
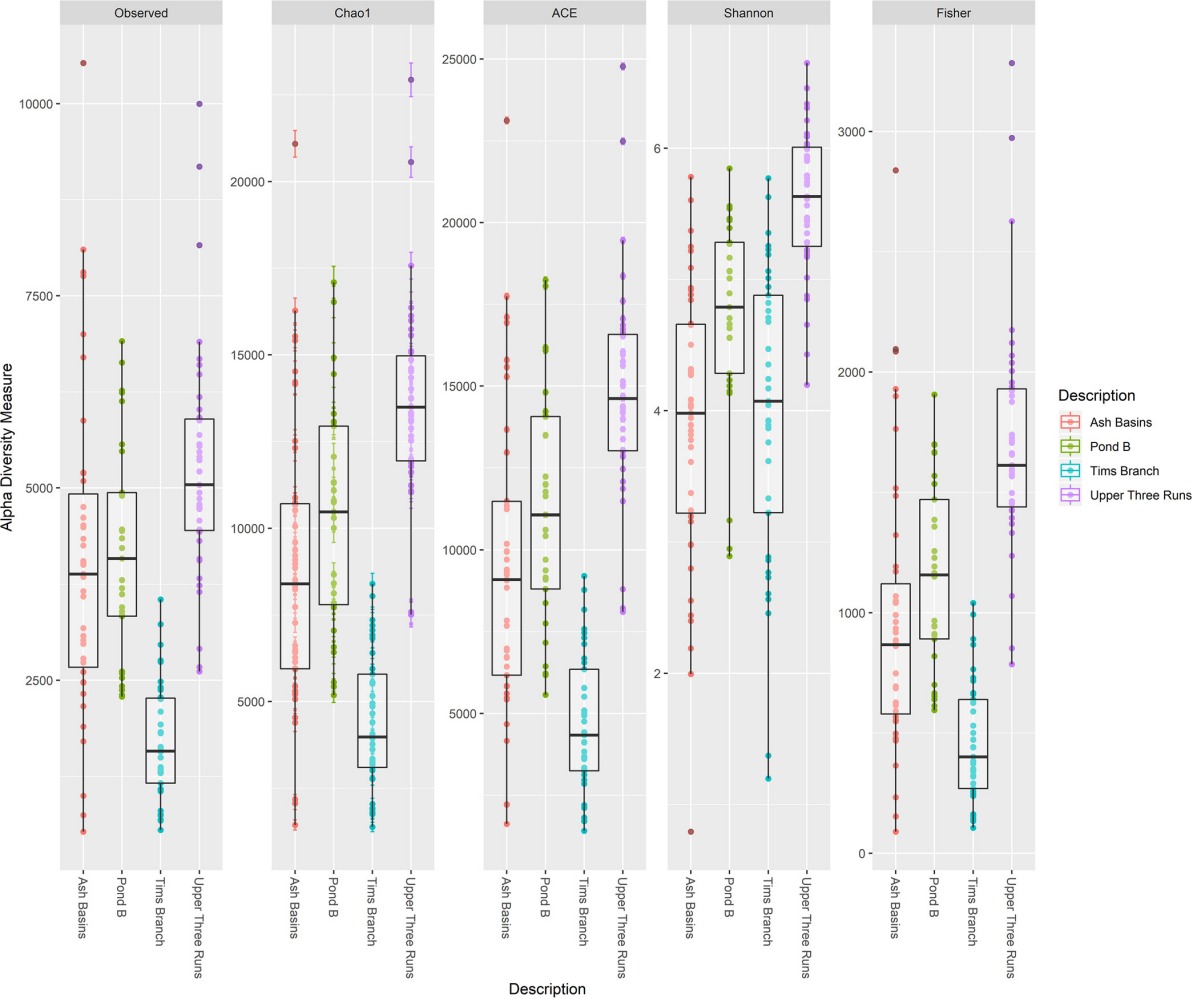
Alpha diversity measures for bacterial taxa in all Peromyscus gossypinus intestinal samples (small and large) at the four sites (defined either by the number of bacterial/archaeal OTUs observed or by Chao1, ACE, Shannon, inverse Simpson, and Fisher diversity measures).

Analysis of microbial diversity indicated there was significantly reduced species, or OTU richness, in all samples from sites with elevated HMs compared to UR (Fig. 2; see Table S5 in the supplemental material). Pairwise group comparisons using Chao1 and Faith’s phylogenetic diversity also indicated that there were differences between sites, including between the contaminated sites. Notably, samples from PB had higher numbers of unique species than those from AB, with TB samples having the lowest diversity overall (Table S5).

**FIG 3:**
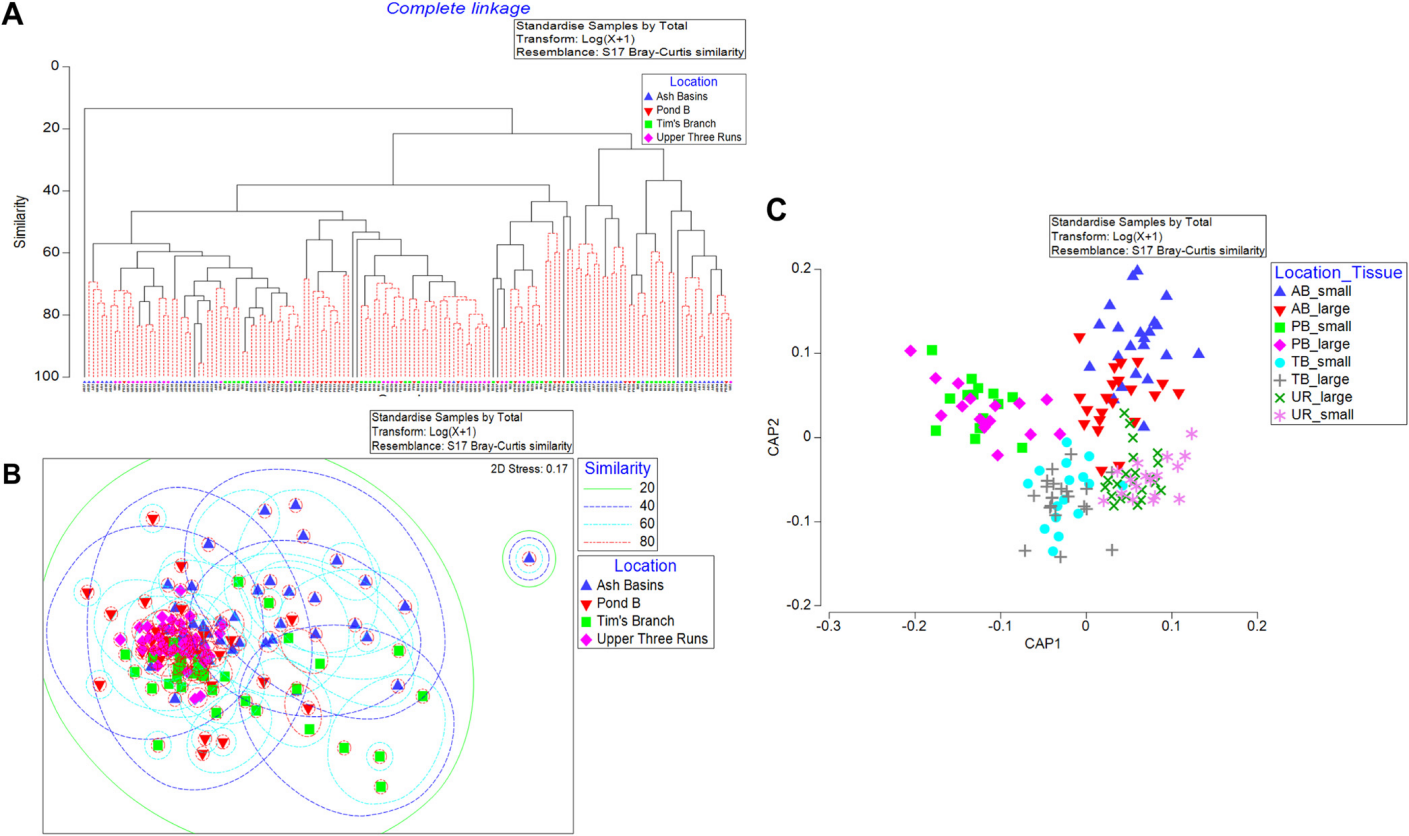
Structure of intestinal microbial communities in Peromyscus gossypinus mice trapped at the Savannah River Site. (A) Nonmetric multidimensional scaling plot of bacterial/archaeal OTU frequency after log transformation, which reduces the influence of the most abundant OTUs. Dashed lines represent percentage of similarity of clusters using SIMPROF: green lines, 20%; dashed blue lines, 40%; dashed cyan lines, 60%; and dashed red lines, 80%. (B) Canonical analysis of principal coordinates based on a Bray-Curtis dissimilarity matrix of log-transformed bacterial/archaeal OTU frequencies. (C) Distance-based redundancy analysis (dBRDA) representing raw Pearson correlations for habitat variables and bacterial/archaeal OTUs. Vectors are overlaid to represent the different HMs and factors most important to the modeling approach. The length and direction of vectors indicate the strength and direction of the relationship. Fitted variation refers to variance within the linear model created during the DistLM analysis. The total variation refers to the variance within the original data. Blue triangles represent samples from Ash Basins (AB), red upside-down triangles represent samples from Pond B (PB), green squares represent samples from Tim’s Branch (TB), and pink diamonds represent samples from the Upper Three Runs (UR).

Unconstrained nonmetric multidimensional scaling (NMDS) plots based on a Bray-Curtis dissimilarity matrix were overlaid with similarity profile routine (SIMPROF)-based cluster analysis data to examine overall similarity between sites in a multidimensional space (Fig. 3A). Overall, the NMDS plots indicated the GMCs displayed between 20 and 40% similarity; however, the similarities within groups showed a high degree of variability (Fig. 3B). To unravel patterns in multidimensional space that might be masked by high variability and high correlation structure among unrelated variables, we also applied canonical analysis of principal coordinates (CAP). The results of the CAP-constrained ordination demonstrated that both the first squared canonical correlation (σ_1_^2^ = 0.9218) and second squared canonical correlation (σ_1_^2^ = 0.8906) were large, indicating the significance of the CAP model. Both canonical axes showed distinct separation of the samples based primarily on the sampling origin of the mice but also according to the intestinal tissue type (small versus large) (Fig. 3C). In addition, multivariate analyses based on the Bray-Curtis distances between 16S rRNA profiles revealed that the type of intestinal tissue had a significant effect on the observed OTUs between samples (permutational analysis of variance [PERMANOVA], pseudo-*F* = 2.6363, *P* = 0.001). Similarly, the site of origin for the mice had a significant effect on the observed OTUs regardless of the type of intestinal tissue (PERMANOVA, pseudo-*F* = 9.3664, *P* = 0.001) (see Table S6 in the supplemental material). Our results also revealed that the sex of the mice had no significant effect on the clustering of samples (PERMANOVA, pseudo-*F* = 1.1628, *P* = 0.208).

### Predicting antibiotic and metal resistance genes from 16S rRNA data using PICRUSt.

For the 171 intestinal samples, the weighted mean of the nearest sequenced taxon index (NSTI), which measures the prediction accuracy of PICRUSt, was 0.15 ± 0.07, which is similar to a previous study on soils (41). When examining the proportion of sequences belonging to signaling pathways that confer resistance to HM contamination, we found significant differences in the pathway categories “Xenobiotics Biodegradation and Metabolism” (*P* = 3.82e−5, false-discovery rate [FDR] corrected) and “Membrane Transport” (*P* = 1.04e−4) (Table S6). We also examined the relative abundance of several KEGG orthologs (KOs) that correspond to antibiotic resistance genes (ARGs) or metal resistance genes (MRGs) to examine differences between sampling sites (see Table S8 in the supplemental material). Several of these KOs, such as TC.HME (K07239; heavy metal exporter), *mdtB* (K07788; multidrug pump), *chrA* (K07240; chromate transporter), and copper resistance genes *cusS* (K07644) and *cusA* (K07787), among others, were significantly enriched (*P* < 0.01, FDR corrected) in mice from the contaminated sites compared to the reference (see Tables S9 and S10 in the supplemental material). Based on the 16S rRNA data, the PICRUSt script *metagenome_contributions.py* revealed that taxa such as Pseudomonas spp., *Epulopiscium* spp., *Adlercreutzia* spp., *Ruminococcus* spp., *Desulfovibrio* spp., and Treponema spp. contributed the greatest number of matched reads for several predicted ARG- and MRG-like genes (see Table S11 in the supplemental material).

### Metagenome sequencing and assembly summary.

For the 24 samples from the four sites, we obtained 2.94 × 10^8^ high-quality reads from the mouse gut shotgun libraries, which were assembled into 1.14 × 10^8^ contigs with an average contig length of 332 bp (see Tables S12 and S13 in the supplemental material). The assemblies contained 4.00 × 10^5^ open reading frames (ORFs) with an average length of 139.66 bp (Table S13). We identified 7.96 × 10^3^ antibiotic resistance gene (ARG)-like ORFs using the SARGfam v.2.0 database and 4.55 × 10^3^ antimicrobial/biocide efflux and metal resistance gene (AB-MRG)-like ORFs using the BacMet v.2.0 database (Table S13).

### Differences in gut antibiotic resistance gene abundance between sites.

**FIG 4:**
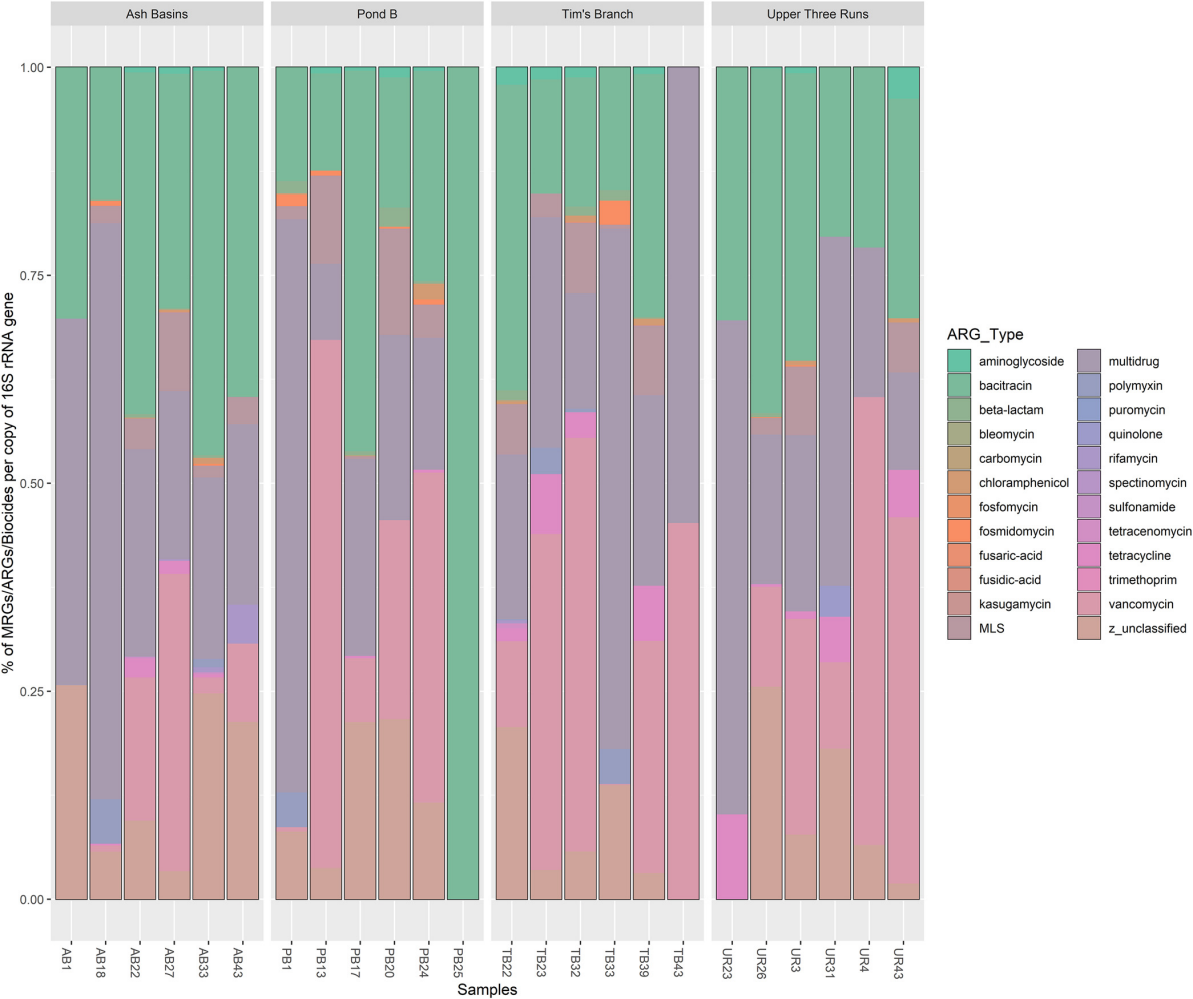
Bar plots showing the relative abundance of BLAST hits for the most abundant ARG types observed in all samples.

In total, we detected an average of 0.13 antibiotic resistance gene (ARG) per 16S rRNA copy, with the predominant ARG types consisting of multidrug resistance genes (37%), bacitracin resistance (24%), vancomycin resistance genes (17%), and unclassified (12%) (Fig. 4; see Table S14 in the supplemental material). The mean abundance of ARG types was lowest (mean ± SD ARGs per 16S rRNA copy) at UR (0.10 ± 0.06 ARGs), whereas ARGs were highest at TB (0.19 ± 0.19), followed by PB (0.17 ± 0.16) and AB (0.10 ± 0.6) (see Table S15 in the supplemental material). We did not detect statistically significant differences in the abundance of ARG types between sites or the particular class of antimicrobials.

**FIG 5:**
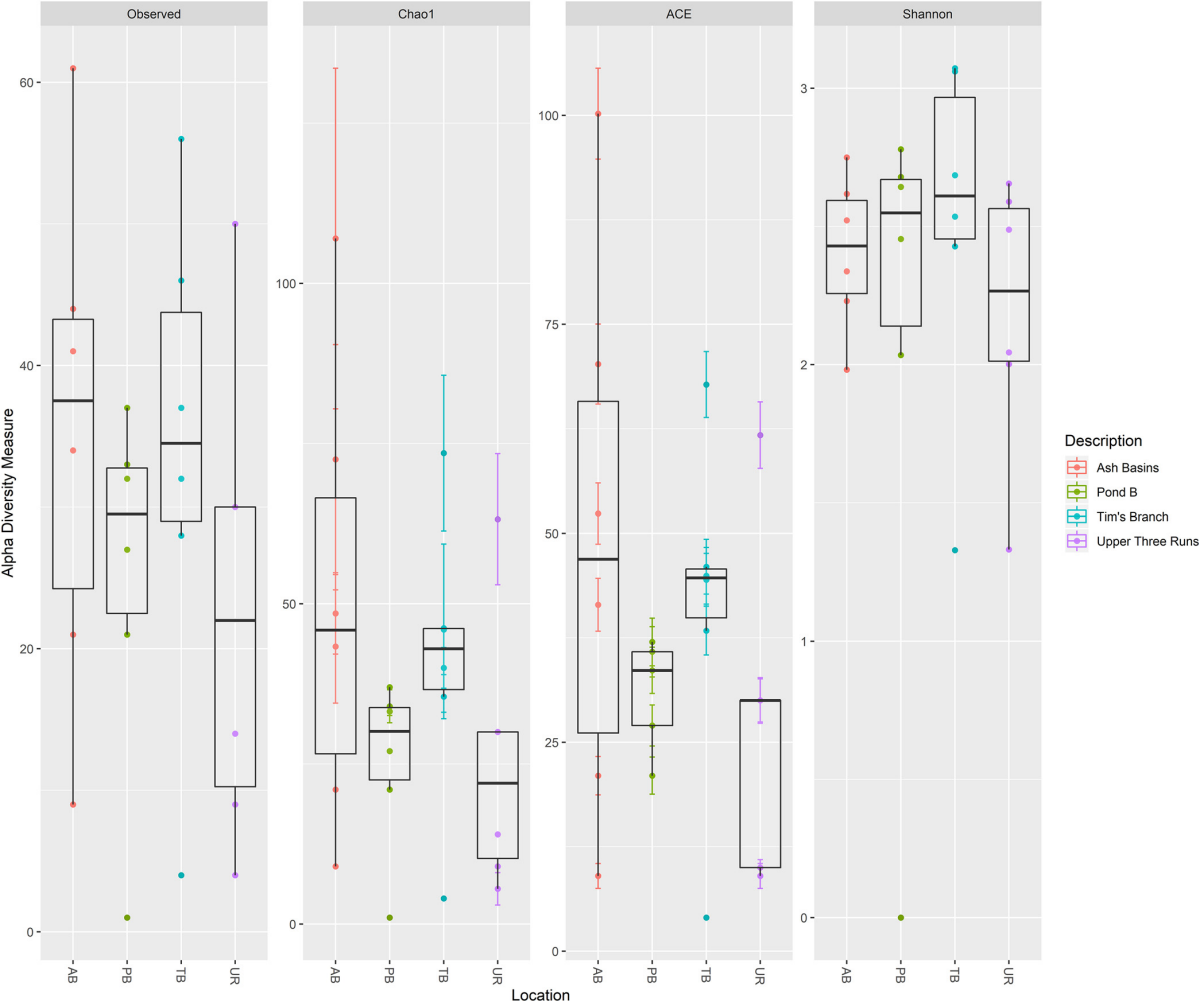
Alpha diversity measures of detected ARGs (based on SARGfam) in all Peromyscus gossypinus intestinal samples (small and large) at the four sites (defined either by the number of bacterial/archaeal OTUs observed or by Chao1, ACE, Shannon, inverse Simpson, and Fisher diversity measures).

We also looked at ARG subtypes, or the genes belonging to a class of ARGs, to determine which specific ARG subtypes were present in the mouse gut samples. The majority of detected ARG subtypes included those that belong to two-component and multidrug transporters. The bacitracin resistance gene *bacA*, vancomycin resistance gene *vanR*, and the multidrug resistance gene *mexT* were among the top three most abundant subtypes. Other ARGs detected in the top 20 ARG subtypes included several multidrug resistance genes from the resistance-nodulation-division (RND) family of transporters, such as *mexF*, *mexT*, *mdtB*, *mdtC*, and *oprN*, among others. We also detected ARG subtypes, including *lsa*, an ATP-binding cassette (ABC) transporter gene that confers resistance to macrolide-lincosamide-streptogramin B (MLS) compounds. Also, we detected the *arnA* gene, which allows Gram-negative bacteria to resist cationic antimicrobial peptides such as polymyxin, and several vancomycin resistance genes in the *van* cluster of genes (*vanG*, *vanS*, *vanX*, and *vanY*). Shannon indices between gut samples with at least two ARG types indicated ranges between 1.32 and 3.07, with TB and AB samples displaying higher indices on average than the reference site (Fig. 5; see Table S16 in the supplemental material).

### Differences in gut antimicrobial/biocide efflux and metal resistance gene abundance between sites.

**FIG 6:**
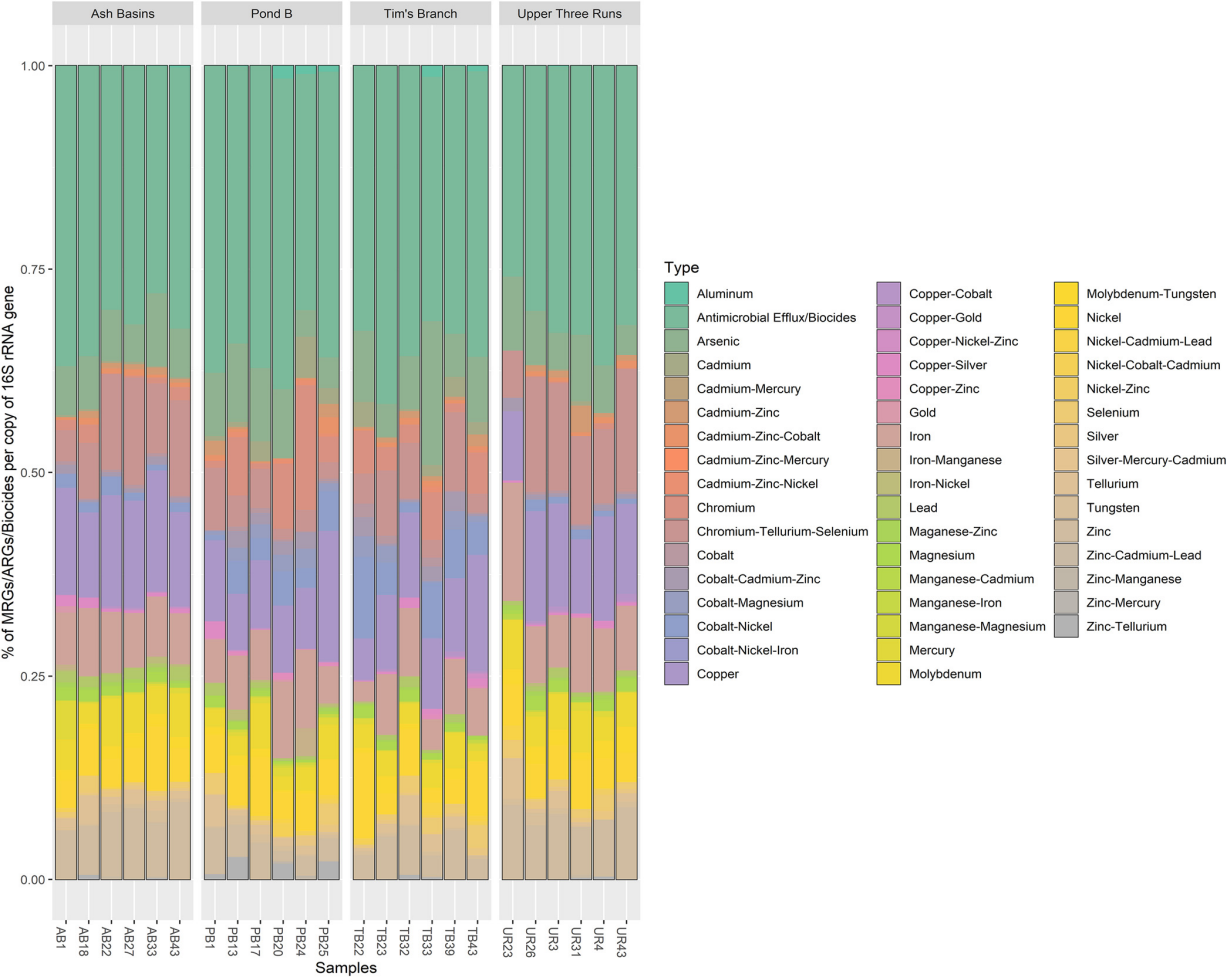
Bar plots showing the relative abundance of BLAST hits for the most abundant AB-MRG types observed in all samples.

**FIG 7:**
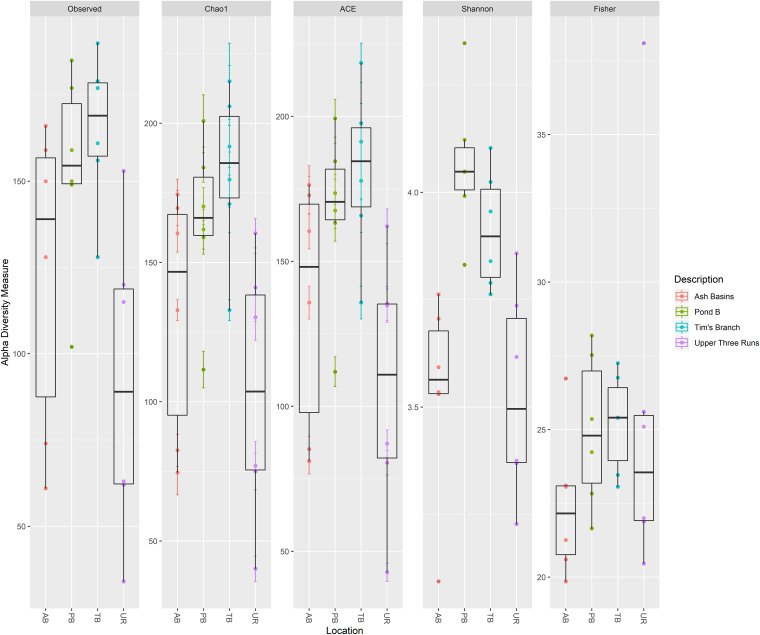
Alpha diversity measures of detected ARGs/biocides (based on BacMet v.2.0) in all Peromyscus gossypinus intestinal samples (small and large) at the four sites (defined either by the number of bacterial/archaeal OTUs observed or by Chao1, ACE, Shannon, inverse Simpson, and Fisher diversity measures).

To assess the presence and diversity of genes associated with antibiotic, biocide, and metal resistance (AB-MRGs), we used the BacMet v2.0 database (see Table S17 in the supplemental material). Our results indicated that the predominant resistance type included several resistance genes in the antimicrobial/biocide efflux category (34%), such as *fabl*, *phoB*, *emrB*, and *baeR* (Fig. 6; see Table S18 in the supplemental material). The relative abundance of antimicrobial/biocide efflux genes varied per sample, but the number of resistance genes was significantly higher (average ± SD ARGs/biocides per 16S rRNA copy) in samples from PB (9.10 ± 19.02) and TB (19.06 ± 41.40) compared to UR (0.26 ± 0.10) and AB (0.21 ± 0.11) (see Table S19 in the supplemental material). Shannon indices also showed that ARG subtype diversity, which ranged from 3.10 to 4.35, was significantly higher among mouse gut samples from PB and TB than those from the UR reference site and AB (Fig. 7; see Table S20 in the supplemental material).

**FIG 8:**
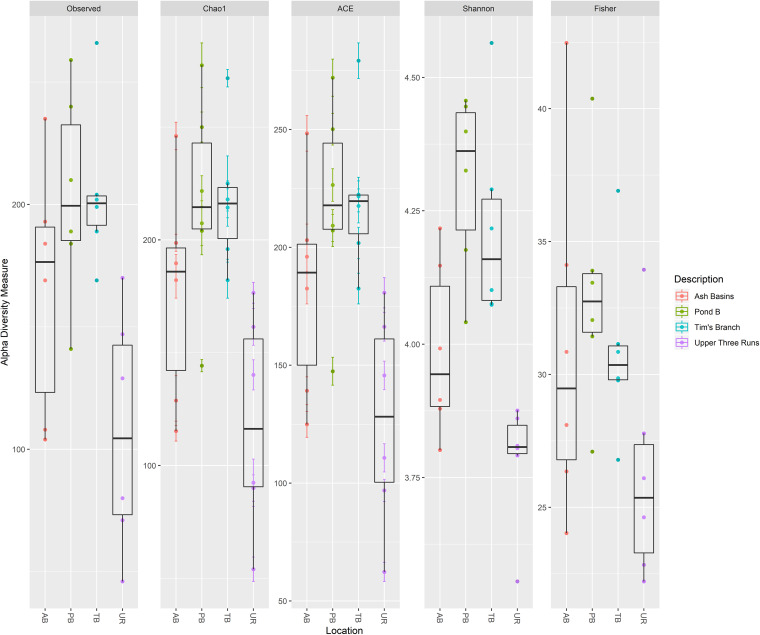
Alpha diversity measures of detected MRGs (based on BacMet v.2.0) in all Peromyscus gossypinus intestinal samples (small and large) at the four sites (defined either by the number of bacterial/archaeal OTUs observed or by Chao1, ACE, Shannon, inverse Simpson, and Fisher diversity measures).

We observed that the predominant metal resistance gene (MRG) subtypes were among several classes of membrane transporters, including ABCs and P-type ATPases (Table S17). Several of the top 50 subtypes confer resistance to one or multiple HMs and included those that confer resistance to Ni (*nrsR*, *dmeR*, and *nikD*), Fe (*furA*, *ideR*, and *bfrA*), Cu (*copR*, *ricR*, and *cusR/ylcA*), Cr-Te (*ruvB* and *recG*), Cd (*actR*), and Co-Ni (*fecE*) (Table S16). The *nrsR* gene was the most abundant MRG subtype overall between all samples (average of 0.34 MRG gene per 16S rRNA copy) and encodes a protein that is part of a two-component signal transduction system involved in Ni^2+^ sensing. In addition, we found significant differences in the abundance of several MRG types, including Cu, As, Ni, Fe, Co-Ni, and Cr, among others (Table S19). Pairwise comparisons revealed that many of these MRG types (e.g., Co, Ni, and Cd-Zn-Ni) were significantly greater (mean ± SD MRGs/biocides per 16S rRNA copy) in abundance in samples from PB (0.33 ± 31.97) and TB (0.67 ± 69.34) compared to either AB (0.01 ± 0.24) or UR (0.01 ± 0.22) (Table S18). Shannon indices based on the MRG subtypes ranged from 3.56 and 4.56, and we observed significantly higher diversity of MRGs in samples from PB and TB than in those from either AB or UR (Fig. 8; see Table S21 in the supplemental material).

### *Peromyscus gossypinus* as host of antibiotics, biocides, and metal resistance genes.

**FIG 9:**
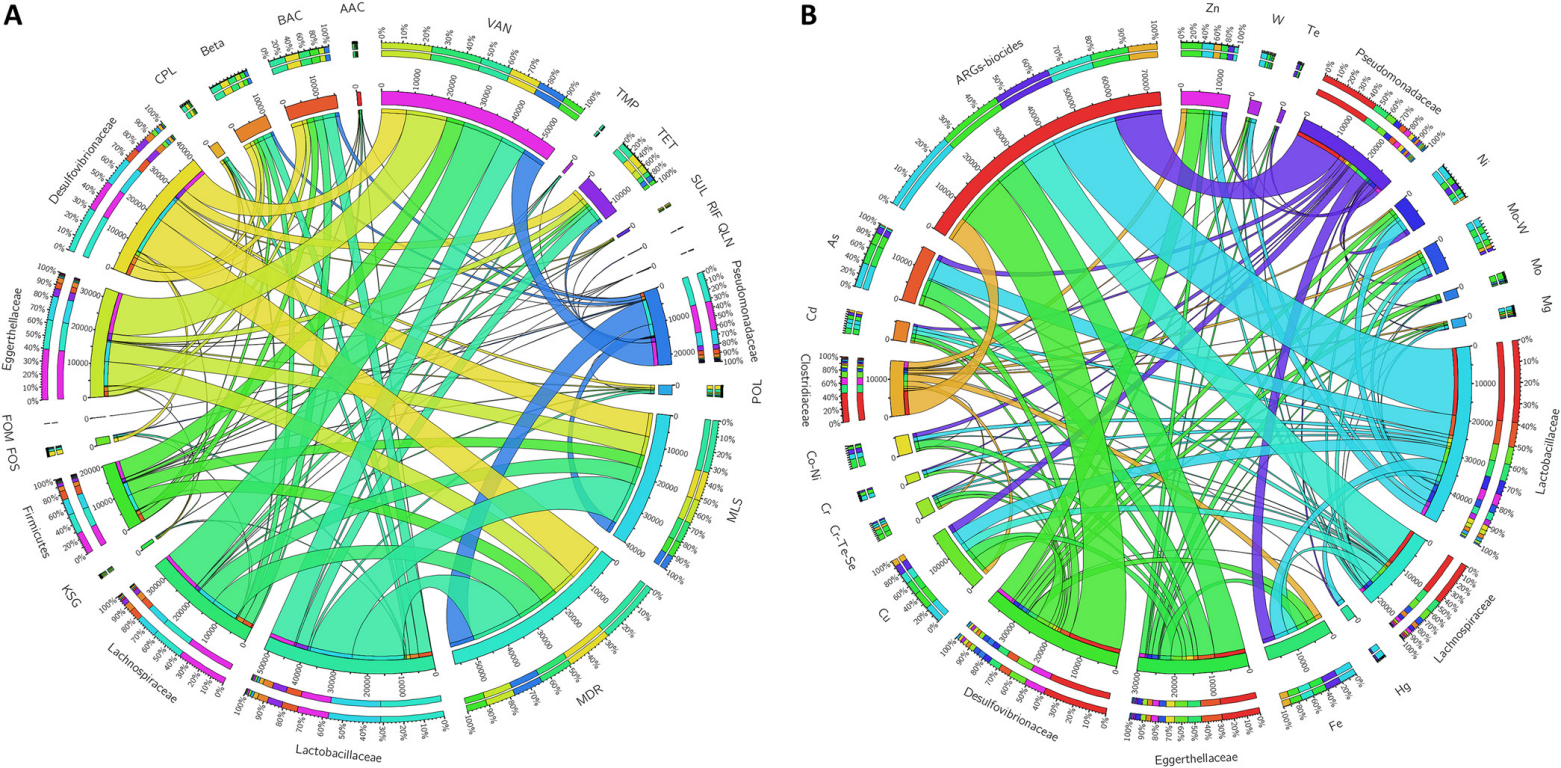
Circos plots displaying percentages of the top ARG-like and MRG-like carrying bacterial hosts. Bars surrounding plot represent the percentage of a particular ARG- or MRG-like gene that was observed in the bacterial hosts. Plots are based on the TPM data of reads mapped to the (A) SARGfam and (B) BacMet v.2.0 databases. Abbreviations are as follows: Act_b, *Actinobacteria*; Act_m, *Actinomadura*; Mycobact, Mycobacterium; Solirubro, *Solirubrobacterales*; MDR, multidrug resistance; TET, tetracycline; AAC, aminoglycoside; MLS, macrolide-lincosamide-streptogramin B; Beta, β-lactam; BAC, bacitracin; CPL, chloramphenicol; FOM, fosfomycin; FOS, fosmidomycin; KSG, kasugamycin; POL, polymyxin; QLN, quinolone; RIF, rifamycin; SUL, sulfonamide; TMP, tetracenomycin; VAN, vancomycin; Ant_Biocides, antimicrobial efflux/biocides; Verru, *Verrucomicrobia*.

From the assembled metagenomes, we detected numerous open reading frames (ORFs) harbored by a diverse collection of taxa; however, we focused on the top six bacterial families as they represented 83% of antibiotic resistance gene (ARG)-like ORFs (SARGfam v.2.0) (Fig. 9A). These bacterial hosts included *Lactobacillaceae*, *Desulfovibrionaceae*, *Eggerthellaceae*, *Lachnospiraceae*, *Pseudomonadaceae*, and *Clostridiaceae*. *Lactobacillaceae*, the largest ARG-like ORF-containing family, possessed resistance genes for the following types of resistance: multidrug (31%), macrolides-lincosamides-streptogramin B (MLS) (26%), vancomycin (16%), bacitracin (8%), tetracycline (7%), and several others (12%). *Desulfovibrionaceae*, the second largest bacterial host possessed several ARG-like ORFs, including those associated with multidrug resistance (29%) and resistance to vancomycin (20%), MLS (18%), bacitracin (9%), and tetracycline (9%). Taxa in the *Eggerthellaceae* family had a predominance of ARG-like ORFs associated with vancomycin (39%), multidrug (23%), MLS (19%), and tetracycline (6%) resistance, while those in the *Pseudomonadaceae* family had a predominance of ARG-like ORFs associated with multidrug (33%), vancomycin (32%), MLS (16%), and tetracycline (6%) resistance.

We observed similar patterns between taxa with respect to antimicrobial/biocide and metal resistance genes (AB-MRGs). Approximately 65% of AB-MRG-like ORFs (BacMet v.2.0) were detected in the top six bacterial taxa and included *Desulfovibrionaceae*, *Lactobacillaceae*, *Eggerthellaceae*, *Pseudomonadaceae*, *Lachnospiracae*, and unclassified *Firmicutes* (Fig. 9B). *Desulfovibrionaceae* possessed resistance genes associated with antimicrobial/biocide efflux (46%) and resistance to Zn (12%), Cu (8%), Fe (7%), As (6%), Ni (4%), and several others. Taxa in the family *Lactobacillaceae* possessed resistance genes for antimicrobial/biocide efflux (43%) and resistance to As (12%), Fe (8%), Ni (7%), and Zn (5%). *Eggerthellaceae* possessed resistance genes for antimicrobial/biocide efflux (48%) and resistance to Fe (9%), Cu (7%), As (7%), Mo (6%), and Ni (4%). Finally, *Pseudomonadacae* possessed resistance genes associated with antimicrobial/biocide efflux (51%), followed by resistance to Fe (13%), Cu (7%), Ni (6%), and As (5%), among several others.

### Co-occurrence of antimicrobial/biocide efflux and metal resistance genes in *P. gossypinus*.

**FIG 10:**
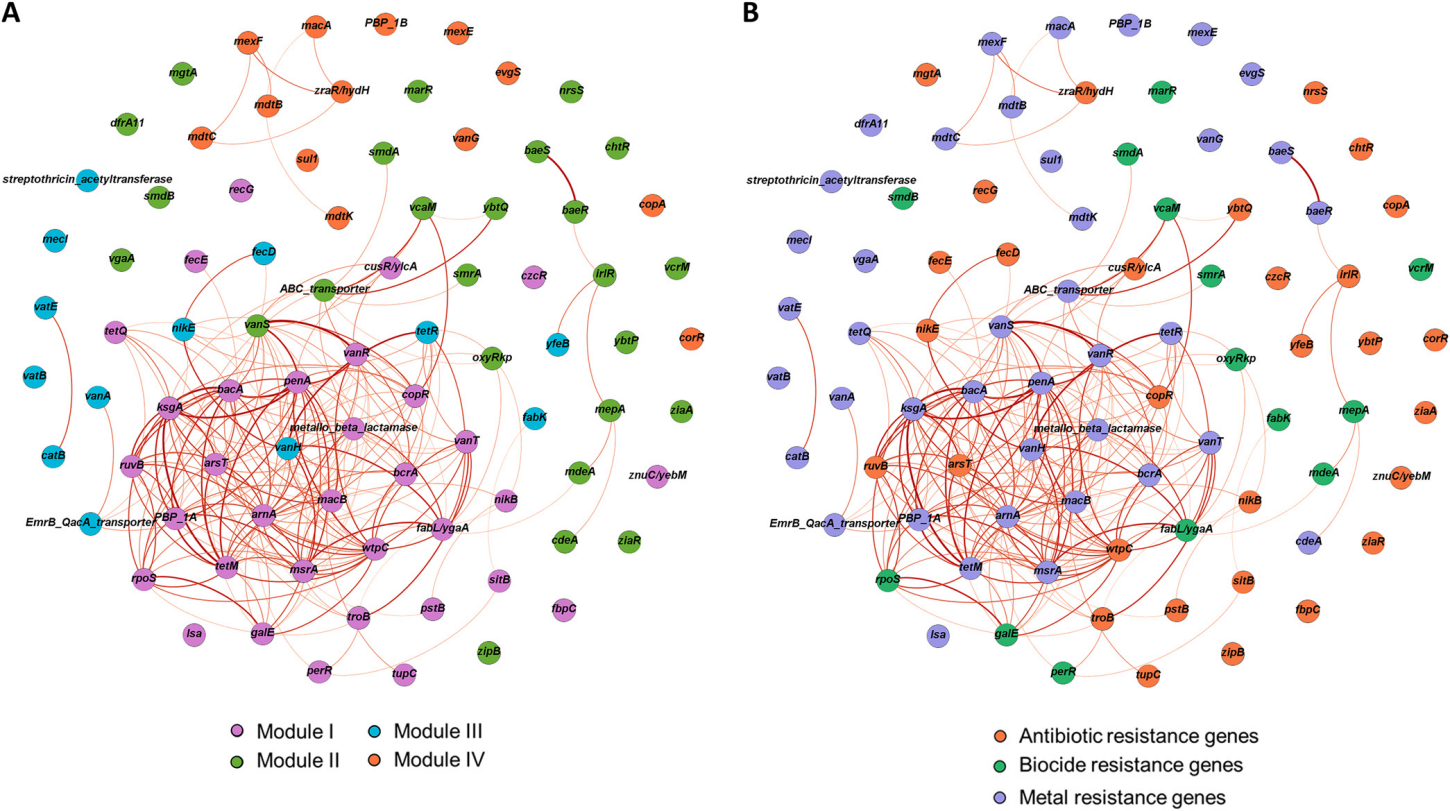
Network analysis showing the co-occurrence patterns of antibiotic, biocide, and metal resistance genes detected in the top 25 taxa. (A) The nodes with different colors represent antibiotic (orange), biocide (green), and metal resistance (light purple) genes. The intensity of edges corresponds to the degree of the positive correlations ranging from 0.61 (light orange) to 0.91 (dark red). (B) The nodes with different colors represent the six modularity classes, with the colors of edges corresponding to their respective class: module I, orange; module II, pink; module III, green; module IV, dark red; module V, cyan; and module VI, light brown. A connection represents a strong Spearman correlation (*P* > 0.6) and significant (*P* < 0.05) correlation (FDR). The size of each node is proportional to the number of connections.

We focused primarily on the predominant (antibiotic resistance gene) ARG-like and (metal resistance gene) MRG-like ORF-containing taxa (*n *= 25) observed in the mouse gut samples to determine patterns of co-occurrence. The resulting network contained 84 nodes and 1,168 edges, which resolved into four modules based on the shared connections within the network (Fig. 10A and B). The largest module (module I) contained 33 subtypes, the majority consisting of ARGs conferring resistance to bacitracin (*bacA*), kasugamycin (*ksgA*), quinolones (*penA* and β-lactamase), vancomycin (*vanH* and *vanR*), macrolides (*macB*), and tetracycline (*tetM*) connected to MRGs conferring resistance to As (*arsT*), Ni (*nikB*), Tu/Mo (*wtpC*), and Pb (*pstB*). This module appeared to be supported by *ksgA* acting as the hub (i.e., a node with large number of connections). Module II, in which *baeS* was the hub, consisted of 25 subtypes, including genes conferring multidrug resistance (*mepA*) or resistance to vancomycin (*vanS*), biocides (*marR*), Fe (*ybtQ* and *ybtP*), Ni (*nrsS*), and several others. Module III, in which *vanH* was the hub, contained 13 subtypes, including several ARGs (*tetR*, *vanA*, EmrB_QacA transporter, *vatB*, *vatE*, and *catB*), biocides (*fabK*), and Ni (*nikE*) among others. Module IV, in which *mdtK* was the hub, contained 11 subtypes, including several ARGs (*sul1*, PBP_1B, *vanG*, *mexE*, *mexF*, *mdtB*, and *mdtC*) and resistance genes for Cu (*copA*) and Zn (*zraR*/*hydH*). Analysis of the positive correlations in the co-occurrence network revealed that many ARGs, MRGs, and biocides tended to co-occur more than would be expected by chance when considering resistance gene type/category and random associations. We measured the co-occurrence of these genes using the ratio of the percentage of observed incidences to percentage of random incidences (O/R ratio) (Table S21). For instance, our analysis showed that bacitracin and multidrug ARGs tended to co-occur more than would be expected by chance with each other (O/R = 2.49), as well as with several genes conferring resistance to Mo/Tu and kasugamycin (O/R = 2.98), As (O/R = 1.99), and Co/Ni (O/R = 1.99) (see Table S22 in the supplemental material).

## DISCUSSION

While the processes that shape the gut microbial communities in wildlife species are still poorly understood, recent studies using high-throughput DNA sequencing have begun to illuminate the influence of various factors, such as evolution, seasonality, diet, gut physiology, host sex, environmental differences, and even contamination, on GMCs (42). We present the first analysis that investigates the potential effects of environmental HMs and radionuclide contamination on the GMCs of wild *P. gossypinus* and provide new data exploring the co-occurrence of antibiotic, biocide, and heavy metal resistance genes associated with the microbiome. Although all *P. gossypinus* mice captured shared a core microbiome, we found in agreement with our hypothesis, that mice inhabiting historically contaminated areas possessed GMCs with significantly lower diversity than mice from the uncontaminated reference site. We provide evidence that the gut resistome of *P. gossypinus* is more closely linked to variation in HMs and radionuclide contamination than would be expected by chance.

In the wild, the staple diet of *Peromyscus* spp. encompasses a variety of foods, including insects, roots, nuts, wild seeds, and grains (39, 43). Similar to other small rodents, species of *Peromyscus* possess a rapid metabolism, requiring them to use an optimal foraging strategy to meet their high energy demands (39, 43). This diet is reflected in the gut of wild *P. gossypinus*, which harbors diverse microbiota that can exploit a variety of dietary substrates. We observed a core microbiome consisting of high abundances of *Firmicutes*, *Proteobacteria*, *Actinobacteria*, *Bacteroidetes*, and TM7 across all study sites. These results are largely consistent with other observations on the GMCs in other wild mammals, particularly other rodents (6, 44–51). However, within this group, we noted the percentage of *Proteobacteria* was particularly high (26.85% ± 0.30%), and there were noticeably lower number of reads assigned to *Bacteroidetes* (6.41% ± 0.03%). Notably, our study contrasts with several previous studies on the GMCs of captive laboratory house mouse strains and wild house mice (e.g., Mus musculus and Mus domesticus, respectively), which have frequently reported a microbiome codominated by the phyla *Firmicutes* and *Bacteroidetes* (6, 45, 52–54). There are fewer studies on the GMCs of *Peromyscus*, a distant relative to mice in the *Mus* genus, but previous work has indicated largely similar patterns at the phylum level (49, 55). Our analyses were most similar to a recent study on wild deer mice (Peromyscus maniculatus), where *Firmicutes* were the dominant phylum, followed by *Bacteroidetes* and then *Proteobacteria* (44). From that study, it was also shown that deer mice raised in captivity have GMCs with a *Firmicute*-*Bacteroidetes* dominant enterotype and significantly less *Proteobacteria* compared to wild mice (44, 56, 57). Nonetheless, differences between the GMCs observed in the *P. gossypinus* mice described here and in other studies are likely driven by the microbial metacommunities specific to the SRS environment (i.e., acquired from complex diets, habitats, seasonality, range, etc.) and social interactions within the population (44).

Within each of the dominant phyla, we detected several bacterial families in the GMCs of *P. gossypinus*. The lactic-acid-producing families *Lactobacillaceae*, *Ruminococcace*, and *Lachnospiraceae* are among the most commonly reported members in the phylum *Firmicutes* in humans and wild animals (6, 45, 52–54). These organisms are important in host physiology and are involved in hydrolyzing starch and sugars to produce butyrate and other short-chain fatty acids (58, 59). *Proteobacteria* is the largest and most diverse bacterial phylum, but its role in host GMCs is still poorly understood. In contrast to the strict anaerobes dominating the GMCs, the majority of *Proteobacteria* consist of facultative or obligate aerobes (60). It has been hypothesized this group plays a key role in successive colonization by the strict anaerobes in the neonatal gut, particularly following postpartum, when there is an abundance of oxygen (60). Proteobacteria have previously been shown to contribute the most functional variation in the human GMCs, possessing the capacity to metabolize a range of organic compounds, including proteins, carbohydrates, and lipids (60, 61). In humans and murine models, high abundances of *Proteobacteria* have been associated with dysbiosis in hosts with metabolic or inflammatory disorders (62–64). However, considering data from a previous study on *P. gossypinus*, there is little evidence that suggests overt health abnormalities in the trapped mice or abnormal mouse mortality or morbidity in the greater mouse population at the SRS, even in mice from historically contaminated areas (35). Instead, our analyses provide additional evidence that *Proteobacteria* are a common feature of wild mice with highly diverse GMCs (52, 54, 65). The families *Desulfovibrionaceae*, *Pseudomonadaceae*, and *Helicobacteraceae* were dominant *Proteobacteria* in wild cotton mice. *Desulfovibrionaceae*, a family of sulfate-reducing bacteria (SRB), have been previously observed in mice fed with high-fat diets (66, 67). Interestingly, there is evidence that SRB members in the *Desulfovibrionaceae* family can tolerate high radiation levels and possibly have utility in the bioremediation of HMs and radionuclides, which suggests a potential detoxifying role in the wild cotton mouse (50, 51). Similarly, *Pseudomonadaceae* contains several members with the capacity for HM and radionuclide bioremediation due to their ability to form biofilms (57, 58). In addition, although *Helicobacteraceae* contains several opportunistic pathogens, the literature suggests it is a common feature of wild murine GMCs (44, 49, 68, 69). The presence of *Helicobacter* strains reinforces the idea that wild murine populations can potentially act as reservoirs of pathogenetic and zoonotic hosts. Indeed, our analyses identified several other known or opportunistic pathogens in relatively low abundance, all within the *Proteobacteria* phylum, including Pseudomonas, Acinetobacter, *Bartonella*, and Klebsiella. Finally, we observed that families in lower abundance (≤5%) included *Coriobacteriaceae* (*Actinobacteria*), S24-7 (*Bacteroidetes*), and F16 (TM7). *Coriobacteriaceae* are important in the conversion of bile salts and steroids, and S24-7 have demonstrated the capacity to degrade complex carbohydrates (70–72). Unfortunately, information on the functional contributions of F16 in the murine gut is currently lacking.

There are limited reports on the impact of radionuclide or heavy metal (HM) exposure on the composition and diversity of gut microbial communities in animals; however, not all studies are in agreement. A study on fecal gut microbiota from wild bank voles (Myodes glareolus) in the Chernobyl Exclusion Zone indicated a shift from *Bacteroidetes* to a more diverse *Firmicutes-*dominated GMC in radionuclide-contaminated areas compared to reference sites (51). A similar study on wild bank voles reported no significant effects of radionuclide exposure on the microbial diversity of GMCs from cecum samples (73). Studies that have looked specifically at HM exposure, appear to overwhelmingly suggest that HMs can alter GMC composition and may reduce overall diversity (45, 55, 74, 75). For example, Richardson et al. (2018) observed that exposure to specific HMs caused perturbations in GMCs, with a noticeable decrease in S24-7 (*Bacteroidetes*) and marked increases in *Proteobacteria*, while Li et al. (2017) reported significantly reduced diversity in mouse GMCs following exposure to cadmium (Cd) in drinking water (75, 76). The difficulty with elucidating the role of radionuclides and HMs on GMCs is that they cannot always be assessed purely by examination of compositional changes. Discrepancies between studies, especially those concerned with wild animals, are highlighted by numerous potential confounding factors, including the intrinsic host GMCs, host diet, duration of exposure, route of exposure (e.g., oral or skin), environmental microbiota, sample type, sample processing, and numerous others (76).

Our metagenomic analyses provided an unparalleled look into the Peromyscus gossypinus resistome that could not have been achieved with traditional PCR screening of antimicrobial resistance (AMR) genes. This was particularly important given that prior studies have demonstrated that rodents, both urban and wild, are involved in carriage of many AMR bacteria (6). Using the SARGfam v.2.0 database, we showed that genes that confer multidrug resistance (37%), bacitracin resistance (24%), and vancomycin resistance (17%) genes were most abundant. The relatively high abundance of multidrug resistance genes was expected, as several studies, including our previous work on Savannah River Site (SRS) soils, have confirmed their ubiquity (34, 77–80). Chromosomally encoded multidrug efflux pumps can extrude a number of substates (e.g., antibiotics, biocides, and HMs) and are conserved across many bacterial species (81, 82). Bacitracin interferes with peptidoglycan synthesis by inhibiting the dephosphorylation of lipid carriers. A previous study showed that bacitracin, a common in-feed antimicrobial, was abundantly present in bovine GMCs (83). In addition, our results indicated genes associated with resistance to vancomycin, a “last resort” broad-spectrum antibiotic, was the third most detected antibiotic resistance gene (ARG) type. We previously showed that vancomycin resistance genes were abundantly present in SRS soils, and this might explain, even partially, the source of vancomycin resistance genes in *P. gossypinus* GMCs (34, 84). In fact, while GMCs are distinctive from those detected in the SRS soils, albeit less diverse taxonomically, both share many overlapping ARGs, and our data support the hypothesis that mobile genetic elements (MGEs) may be horizontally exchanged from environmental reservoirs of resistant bacteria to animals and even humans (84–86).

In examining metal resistance genes (MRG) types, we found that mouse GMCs across all study sites contained a diverse collection of genes associated with heavy metals (HMs), such as Cu (23%), As (9%), Fe (9%), and Ni (9%), among others. The nickel resistance gene *nrsR* was the most abundant MRG subtype, followed by *furA* (Fe) and *copR* and *ricR* (Cu). A number of MRG subtypes, such as *ruvB* (Cr-Te-Se) and *dmeR* (Co-Ni), were also detected that confer cross-resistance to multiple HMs. Several of these resistance genes have been reported in prior studies as having direct correlations with β-lactamases, sulfonamides, macrolide-lincosamide-streptogramin B (MLS), and tetracycline resistance genes (87). For example, Fe and Ni resistance genes have also been shown to be significantly associated with antibiotic resistance genes (ARGs), such as genes associated with multidrug efflux, β-lactamases, sulfonamides, macrolide-lincosamide-streptogamin (MLS), tetracycline, aminoglycosides, and vancomycin (74, 80). Indeed, our analyses of co-occurring ARGs/biocide genes suggested many positive correlations with MRGs than would be expected by chance and provide additional insights into the coselection phenomenon in areas with anthropogenic disturbances. Furthermore, in contrast to our analysis of ARGs, the relative abundance and diversity of MRGs were higher in mice gut samples from radionuclide-contaminated sites such as PB and TB than samples from either AB or UR. Interestingly, with respect to TB, our results are consistent with our previous observations of soil metagenomes, in which MRGs in TB soils were significantly elevated (34). It is likely the higher abundance and diversity of MRGs in mouse GMCs are a consequence of environment-specific selection pressures (i.e., background levels of HMs and radionuclides) at TB than any intrinsic differences between mice captured in this study (88). In fact, this pattern seems to suggest the presence of radionuclide contamination has a more important role in the selection of MRGs than HMs alone. One notable and unexpected result was the lack of MRG types in AB mice. We suggest that because of the sporadic dispersion of coal fly ash at AB, the mice we collected may not have had significant HM exposure, to a level that would enable enrichment of their resistome, even if certain soils’ HMs were significantly higher than the reference.

The emergence, dissemination, and maintenance of antibiotic resistance are a major global health threat. Understanding the intersection between the external environment, human activities, and role of animals in driving resistance is thus crucial in identifying appropriate mitigation strategies. Extending work from our previous study on the Savannah River Site, we sought to elucidate the potential coselection of antimicrobial resistance (AMR) in an antibiotic-naive population of wild *P. gossypinus* (84). Our work supports other studies indicating wild mice are reservoirs for several human pathogens with clinical significance and provides new data on the types of co-occurring antibiotic, heavy metal, and biocide resistance genes associated with wild *P. gossypinus* gut microbial communities (GMCs). Importantly, our work illustrates the necessity of AMR surveillance of GMCs in wild populations, especially in areas with a history of contamination and/or proximity to humans. The available literature suggests many antibiotic resistance genes (ARGs) and metal resistance genes (MRGs) originate from environmental reservoirs, but can be further enriched by anthropogenic pollution (84). Mobile genetic elements can facilitate the spread of these environmentally derived resistance genes to animals, which in turn, may harbor emerging and/or zoonotic bacterial pathogens of human concern.

There are several limitations to this study. Use of 16S rRNA amplicons for microbiome analysis is widely known to introduce PCR primer biases and possesses limited resolution beyond the genus level. While shotgun metagenomics does not suffer from these limitations, genomic DNA extraction, library preparation methods, and even sequencing methodology may preferentially select certain taxa over others. The annotation of metagenomes is largely dependent on the reference database used, often heavily biased toward readily cultivable model organisms, and hence may not capture the total diversity of ARGs or MRGs in a particular environment. Finally, for certain genes or partial matches, their role in antimicrobial and/or metal resistance remains largely speculative due to fewer experimental studies. Therefore, caution must be taken in interpretation of their functional roles.

## MATERIALS AND METHODS

### Mouse trapping and sample collection.

Mice were previously captured from March to mid-May at the SRS, SC, USA, using Sherman live traps (H. B. Sherman Traps, Inc., Tallahassee, FL) baited with black oil sunflower seeds (40). All trapping, handling, and euthanasia practices were approved by the U.S. Army Public Health Command’s Institutional Animal Care and Use Committee. Following dissection, mouse intestinal samples were used for DNA extraction, 16S rRNA gene amplicon, and shotgun metagenomic sequencing. Mouse kidney and livers were also analyzed for presence of heavy metals using U.S. EPA microwave-assisted digestion method 3052, but were below the minimum detection limit (MDL) for all metals (Cr, 0.30; Co, 0.05; Ni, 0.07; Zn, 0.45; As-2, 1.65; Sr, 0.15; Pb, 0.03; U, 0.06). Background HMs in the mouse environments (soil) during the time of sampling are as follows: strontium (Ash Basins, up to 176.27 mg kg^−1^ [40.43 ± 49.24 mg kg^−1^]; Tim’s Branch, up to 37.90 mg kg^−1^ [25.05 ± 13.10 mg kg^−1^]) and cobalt (Ash Basins, up to 18.17 mg kg^−1^ [4.53 ± 5.10 mg kg^−1^]; Tim’s Branch, up to 12.99 mg kg^−1^ [5.71 ± 3.38 mg kg^−1^]) (Table S1).

### Microbiome DNA sequencing, bioinformatics, and data analyses.

From the 86 mice, DNA was extracted from 0.5 g of gut tissue using a MoBio PowerSoil DNA isolation kit (MoBio, Carlsbad, CA, USA) and purified by a magnetic-based size selection method using SeraPure Speedbeads (Thermo-Fisher Scientific, Asheville, NC, USA) according to the manufacturer’s protocol. Dual-indexed PCR libraries for bacterial 16S rRNA gene analyses were generated using the TaggiMatrix protocol as described in our previous study (34, 89, 90). Metagenomic libraries for 24 intestinal samples (six from each location) were prepared using NEB Ultra II FS kits (New England Biolabs, Ipswich, MA, USA) following the manufacturer’s protocol at half-volume reactions with two modifications: 5 μM iTru y‐yolk adaptors during ligation and 5 μM iTru indexed primers during PCR for 9 cycles (84, 85). Both 16S rRNA amplicon and shotgun metagenome libraries were sequenced on an Illumina sequencing platform with MiSeq (PE300) and NovaSeq (PE150), respectively, at the Georgia Genomics and Bioinformatics Core (GGBC, University of Georgia). All subsequent bioinformatic and statistical analysis were conducted using the same software packages, scripts, and workflows described in our previous study (34).

Briefly, paired-end sequencing reads were merged using the FLASH v.1.2.9 plugin, and Mr_Demuxy v.1.2.0 (https://pypi.python.org/pypi/Mr_Demuxy/1.2.0) was used to demultiplex merged reads into individually tagged fastq files based on their internal barcode (91). High-quality reads (≥Q20) were filtered by size (≥400 bp) and used for all subsequent amplicon-based analyses. The software package MacQiime v.1.91 (http://www.wernerlab.org/software/macqiime) was used to identify operational taxonomic units (OTUs), taxonomic classification, and diversity analyses against the Greengenes v.13_8 16S rRNA database and the VSEARCH OTU picking algorithm (92–94). Within-sample (alpha) diversity indices were computed using MacQiime v.1.91 and further visualized using Phyloseq v.3.10 (95). Beta diversity was assessed using a nonparametric Kruskal-Wallis test with the false-discovery rate (FDR) correction for multiple comparisons and the Monte Carlo simulated nonparametric *t* test for pairwise comparisons (96–98). Bray-Curtis distances between sampling locations were computed and visualized using Primer 7.0 software with the PERMANOVA+ add-on (Primer-E, United Kingdom) (99). The software PICRUSt v.1.1 was used to make gene content predictions based on 16S rRNA gene sequences, and selected samples were further examined using shotgun metagenomic sequencing (41). The abundances of ARGs were determined using the ARGs-OAP v.2.0 pipeline, which identifies ARGs against the SARGfam v.2.0 database and performs copy number estimation against essential single-copy marker genes, in addition to the 16S rRNA gene (100). The abundance of AB-MRGs was determined by a hybrid approach, using UBLAST to identify candidate sequences against the BacMet v.2.0 735 experimentally confirmed database (March 2018) (101). The software package Diamond v.0.9.30 (https://github.com/bbuchfink/diamond) was used to perform a BLASTX search on candidate sequences against the BacMet v.2.0 database (102, 103). Spades v.3.13 was used to *de novo* assemble reads using the default *k*-mer size (104). All ORFs were screened using HMMSCAN (https://www.ebi.ac.uk/Tools/hmmer/search/hmmscan) against the SARGfam database (https://github.com/xiaole99/SARGfam) and validated HMM profiles of ARGs (100). The ARG‐like ORFs from the HMM results were extracted from the original protein sequences of ORFs predicted in the contigs using Seqkit v.0.12.0 (https://bioinf.shenwei.me/seqkit/usage/). PROKKA v.1.14 was also used to count and functionally annotate the predicted ORFs, using the SARGfam HMM profile as input (105). Similarly, the MRG‐like ORFs were first annotated using BLASTP against the BacMet v.2.0 database and then annotated using PROKKA against the BacMet v.2.0 database. Bowtie2 v.2.3.5.1 was used to map all sequencing reads to the extracted ARG-like or MRG-like ORF nucleotide sequences of each respective sample group. SAMtools (http://samtools.sourceforge.net/) was used to convert the SAM files to BAM format and to sort by alignment coordinate. Reads that mapped to the assembly were counted using the *prokkagff2gtf* and htseq‐count scripts (106). Abundance values for genes were normalized to predicted transcripts per million (TPM) using *tpm_tably.py*, which calculates the TPM based on average read length and length of gene. Diamond v.0.9.30 was used to annotate the ORFs, by conducting a BLASTP search against the NCBI NR database (downloaded on 29 May 2019) at an E value of ≤10− 5 (102, 103). The diamond BLASTP results were “meganized” and annotated using MEGAN (MEtaGenome ANalyzer, v.6.16.4) taxonomic assignment, using default parameters (voting score of ≤50%) (107). Major bacterial hosts of ARGs and MRGs shared between sites were visualized using Circos Table Viewer v.0.63‐9 (http://mkweb.bcgsc.ca/tableviewer/) (108). Network analyses were conducted by generating a sparse matrix based on combined TPM data from the SARGfam and BacMet databases, filtered by the top 25 most abundant taxa. A co-occurrence network was generated using custom scripts (https://github.com/RichieJu520/Co-occurrence_Network_Analysis) (109). The resulting network was visualized in Gephi v.0.9.3 (110). The R package v.3.6.3 was used to conduct one‐way analysis of variance (ANOVA) or Kruskal–Wallis one‐way analysis of variance, depending on pass or failure of a Shapiro-Wilk test of normality, to compare differences in relative abundance of ARG or MRG number per 16S rRNA copy, in addition to assessing differences in Shannon index. For network analyses, the ratios of the percentage of observed to percentage of random incidences (O/R ratios) of co‐occurrence patterns between antibiotics, biocides, and metals were determined using methods previously described (107, 109, 110). Additional scripts are provided in the supplemental material.

### Data availability.

Copies of high-resolution figures (600 dpi) are available at: https://figshare.com/articles/figure/Figures_for_Wild_Peromyscus_gossypinus_SRS_study/14175554. All 16S rRNA gene and raw metagenomic sequences are available through NCBI under BioProject accession no. PRJNA707221.
