# Supplementary material for: Unveiling the Gut Microbiota and Resistome of Wild Cotton Mice, Peromyscus gossypinus, from Heavy Metal- and Radionuclide-Contaminated Sites in the Southeastern United States
Source: Microbiol Spectr. 2021 Aug 25;9(1):10.1128/spectrum.00097-21. doi: 10.1128/spectrum.00097-21 (PMC8552609; doi:10.1128/spectrum.00097-21)
Supplement: SUPPLEMENTAL FILE 2 — Supplemental material. Download SPECTRUM00097-21_Supp_2_seq12.docx, DOCX file, 1.5 MB [file spectrum00097-21_supp_2_seq12.docx]

**Supplementary Information**


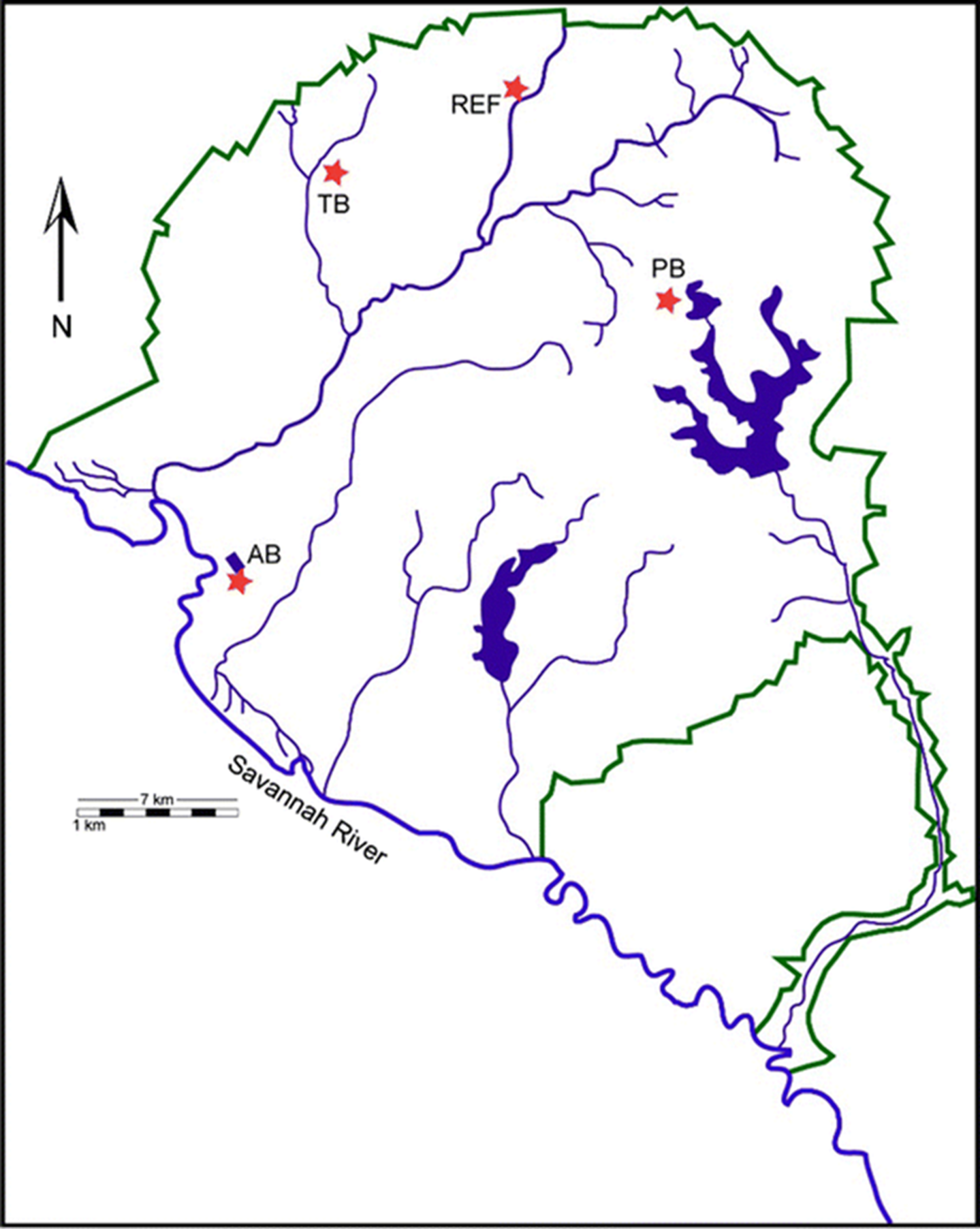


**Supplementary Figure 1**. Map of the Savannah River Site, South Carolina, USA, depicting the distribution of the reference location (Upper Three Runs – UR) and contaminated study areas, Ash Basin (AB), Pond B (PB), and Tim’s Branch (TB) sampled for *Peromyscus gossypinus* in spring 2014

**Hybrid UBLAST and BLASTX methods based on original article by (Yang *et al.*, 2014)**

**Quick search for candidate sequences from MRG database using USEARCH *ublast* command** (Edgar, 2010)**:**

ls *.fa | parallel –gnu “usearch -ublast {} -db “your_database.fasta” -evalue 1e-5 -accel 0.5 -blast6out {/.}.ublast_output_file.txt -threads 4"

**Extract sequence ids contained in column 1 from candidate sequence output files from *ublast***

awk '{if(FILENAME!=prev){close(prev)};print $1 > (FILENAME".ids.txt");prev=FILENAME}' *.txt (or whatever file name is from previous step)

**Extract ublasted candidate sequences from original fastas** <https://bioinf.shenwei.me/seqkit/usage/>**:**

parallel 'cat {} | seqkit grep -f {.}.ids.txt > {.}.output.fa' ::: *fa

**Create a protein database for use with blast:**

makeblastdb -in BacMet2_EXP_database.fasta -dbtype prot -input_type fasta -out db/blast/BacMet2_aa -hash_index

**Create a protein database for use with Diamond blastx** (Buchfink *et al.*, 2015)**:**

diamond makedb --in BacMet2_EXP_database.fasta -d BacMet2

**Diamond blastx all candidate sequences originally derived from USEARCH ublast:**

ls *.fa | parallel --gnu "diamond blastx -q {} -d …/BacMet2.dmnd --max-target-seqs 1 --threads 4 --evalue 0.000001 -o {/.}.diamond.tab"

awk '{if(FILENAME!=prev){close(prev)};print $1 > (FILENAME".diamond_blastx_ids.txt");prev=FILENAME}' *.tab

**Extract final target sequences:**

awk '{if(FILENAME!=prev){close(prev)};print $1 > (FILENAME".ids.txt");prev=FILENAME}' *.tab

**Get counts of each gene per read**

ls *.tab | parallel --gnu "cut -f 2 {} > {/.}.outputfile.txt"

ls *.txt | parallel --gnu "sort {} | uniq -c | sort -n > {/.}.ids_counts"

**Get sequence ids from original BacMet and save to file**

grep '>' …/BacMet2_EXP_database.fasta > file.txt

**Prodigal**

ls *.fasta | parallel prodigal -i {} -o {.}.genes -a proteins{.}.faa -p meta

**Use BBMAP to split large FASTA files for usearch 32-bit version (**<https://jgi.doe.gov/data-and-tools/bbtools/>)

ls *.fasta| parallel --gnu "partition.sh in={} out=out_dir/{/.}part%.fasta ways=5"

**Recombine or concatenate usearch-blastx search results**:

1. touch filename_finish.txt
2. cat file_name_mergedpart* >> filename_finish.txt

**Get counts of ARG or MRG-like contigs:**

wc -l myfile.fa

**Get counts of ARG or MRG-like ORFs:**

MRGs: Grep -c BacMet2 fileNameHere

ARGs: Grep -c Sargfam fileNameHere

**Get ORFs matching to Sargfam or BacMet2:**

grep -nr “Sargfam” PROKKA_05042020.gff > PeroGut_TB_ARG_ORF_ids.txt

**Get annotation ids from gff file (9^th^ field) to use for extraction of the .faa output from Prokka:**

awk '{if(FILENAME!=prev){close(prev)};print $1 > (FILENAME".ids.txt");prev=FILENAME}' *.txt

**Remove text at end of sequence ids:**

awk -F';' '{print $1}' '/directory/prokka_out_ARG/Ids/PeroGut_AB_ARG_ORF_ids.txt.ids.txt' > AB.ids1

Remove ‘=” at beginning and create final sequence id list

awk -F= '{print $NF}' '/directory/prokka_out_ARG/Ids/UR.ids1' > PeroGut_UR_ORFs.ARGs_ids2

awk -F: '{print $NF}' PeroGut_UR_ARGs_ORF_extracted_ids.txt > PeroGut_UR_ARGs_ORF_ids2.txt

**Extract ARG or MRG containing ORFs**

parallel 'cat {} | seqkit grep -f {.}_ids2 > {.}.ARGs.ffn' ::: *ffn (or ffa file from prokka)

ls *.bam | parallel 'htseq-count -r pos -t CDS -f bam /directory/bowtie2_out_ARGs/{.}.bam /directory/prokka_out_ARG/prokka/{.}.gtf > {.}.count'

**References:**

Buchfink, B., Xie, C., and Huson, D.H. (2015) Fast and sensitive protein alignment using DIAMOND. *Nat Methods* **12**: 59–60.

Edgar, R.C. (2010) Search and clustering orders of magnitude faster than BLAST. *Bioinformatics* **26**: 2460–2461.

Yang, Y., Jiang, X.-T., and Zhang, T. (2014) Evaluation of a Hybrid Approach Using UBLAST and BLASTX for Metagenomic Sequences Annotation of Specific Functional Genes. *PLoS ONE* **9**:.
